# Gender-modulated influence of BDNF concentration and Val66Met polymorphism on cognitive outcomes in chronic limb ischemia patients

**DOI:** 10.3389/fendo.2024.1417292

**Published:** 2024-11-15

**Authors:** Aleksandra Chyrek-Tomaszewska, Alicja Popiołek, Agnieszka Stachowicz-Karpińska, Jacek Budzyński, Katarzyna Linkowska, Mariusz Kozakiewicz, Adam Szelągowski, Alina Borkowska, Maciej Bieliński

**Affiliations:** ^1^ Chair and Department of Clinical Neuropsychology, Nicolaus Copernicus University in Toruń, Collegium Medicum in Bydgoszcz, Bydgoszcz, Poland; ^2^ Department of Vascular and Internal Diseases, Nicolaus Copernicus University in Toruń, Collegium Medicum in Bydgoszcz, Bydgoszcz, Poland; ^3^ Department of Forensic Medicine, Division of Molecular and Forensic Genetics, Nicolaus Copernicus University in Toruń, Collegium Medicum in Bydgoszcz, Bydgoszcz, Poland; ^4^ Department of Geriatrics, Nicolaus Copernicus University in Torun, Collegium Medicum in Bydgoszcz, Bydgoszcz, Poland

**Keywords:** brain-derived neurotrophic factor, cognition, gender, genetic polymorphism, val66met, chronic limb ischemia

## Abstract

**Introduction:**

While numerous studies have established associations between brain-derived neurotrophic factor (BDNF) and cognitive functioning, limited research has delved into the role of BDNF concerning cognitive outcomes in atherosclerosis-related conditions. This study aimed to investigate the correlations between cognitive performance, serum BDNF levels, and the BDNF Val66Met polymorphism in individuals diagnosed with chronic limb ischemia (CLI).

**Participants and procedure:**

The study encompassed 159 CLI patients (52 females, 107 males) aged 59-73 years. Genetic analysis involved assessing the BDNF Val66Met single-nucleotide polymorphism using a TaqMan SNP Genotyping Assay and the ViiA™ 7 Real-Time PCR System. Serum BDNF levels were quantified. Cognitive functioning was evaluated through a computerized battery of tests, including the simple reaction test (SRT) for speed and accuracy assessment, verbal memory test (VMT) for short and long-term memory evaluation, and the GoNoGo test for cognitive control and inhibition.

**Results:**

Gender differences in cognitive performance were observed, with women excelling in VMT, while men demonstrated superior performance in SRT and the GoNoGo test. No statistically significant differences were noted between the Met/Met or Met/Val and Val/Val subgroups. However, notable differences emerged in female Met carriers, exhibiting superior VMT scores but more incorrect Go responses in the GoNoGo test. Conversely, female Val homozygotes showed significantly more incorrect NoGo responses compared to male Val homozygotes. In men carrying the Met allele, higher BDNF concentrations correlated with improved GoNoGo test results (r = 0.248, p = 0.01). Conversely, in women with the Val/Val variant, lower BDNF concentrations were associated with better VMT scores.

**Conclusions:**

This study underscores the sex-specific impact of BDNF serum levels and the BDNF polymorphism on cognitive processes among CLI patients. The findings highlight the nuanced influence of BDNF in shaping cognitive functioning, emphasizing the need for further research into these sex-dependent associations.

## Introduction

The brain-derived neurotrophic factor (BDNF) belongs to the neurotrophin family and plays a pivotal role in regulating essential processes like neurogenesis and neuroregeneration (1). Primarily synthesized by neurons, BDNF is prominently present in key brain structures, notably the hippocampal formation and cerebral cortex, which are integral for memory, learning, and higher cognitive functions (2). Although predominantly produced by neurons, lower concentrations of BDNF can be found in non-neuronal cells and are detectable in the periphery (2). BDNF primarily exerts its actions by binding to the tyrosine receptor kinase B (TrkB).

The BDNF is encoded by the BDNF gene, located on chromosome 11, and several of its polymorphisms have been extensively studied in various contexts (3–8) also in the field of cognitive (9) and atherosclerotic issues (10). Among these polymorphisms, a commonly investigated one is a single nucleotide polymorphism (SNP) known as rs6265 or Val66Met. This specific polymorphism involves a point mutation - a substitution of guanine with adenine at position 196 of the pro-BDNF coding sequence. Consequently, this leads to an amino acid exchange from valine to methionine at codon 66 (4). This alteration results in the disruption of BDNF secretion and a reduction in its binding to TrkB receptors (11).

Chronic limb ischemia (CLI) often stands as a significant manifestation of atherosclerosis, a systemic and progressive condition that affects multiple organs throughout the body. In individuals with CLI, various stages of atherosclerosis can concurrently affect cervical or cerebral vessels. This broader systemic involvement of atherosclerosis can have diverse impacts on the brain, leading to conditions such as infarcts, microinfarcts, or cerebral hemorrhages, all of which directly affect brain tissue (12). The progression of atherosclerosis is typically associated with aging. Consequently, apart from age-related changes, neurodegenerative processes such as gray matter shrinkage or the decline in synaptic connections contribute to cognitive deterioration (13, 14). In patients with CLI, both vascular and neurodegenerative dementia are believed to be principal contributors to cognitive impairment.

Research has indicated that BDNF plays a protective role in maintaining the physiological function of vascular endothelium. Decreased serum concentrations of BDNF have been observed in patients affected by atherosclerosis, suggesting a potential link between BDNF levels and the condition (15). This protective effect is believed to occur through the stimulation of stem cells and vascular progenitor cells, the promotion of nitric oxide synthase expression, and the regulation of endothelial oxidative stress, ultimately reducing endothelial damage (16).

Given the established role of BDNF, particularly its Val66Met polymorphism, in the pathogenesis of cognitive decline associated with neurodegeneration, it is plausible to suspect an association between BDNF and vascular cognitive impairment linked to atherosclerosis as well.

## Methods

The assessment encompassed 159 patients diagnosed with Chronic Limb Ischemia (CLI), comprising 52 women and 107 men (but gender was not a criterion for the selection of subjects) selection aged between 59 and 73 years. Demographic and clinical data are located in **Table 1**. According to the descriptive data of the study group, women were statistically significantly older than men. However, no other significant differences were found in terms of demographic and clinical characteristics. Though women had a lower rate of amputation, but this was not statistically significant. These individuals were referred to Jan Biziel’s University Hospital No.2 in Bydgoszcz, Poland, for elective lower limb revascularization (surgical or endovascular) between May 2017 and July 2018. Diagnosis and treatment followed established criteria (17). Prior to their involvement, all patients provided written consent, and the study adhered strictly to the principles outlined in the Declaration of Helsinki. Approval for the research protocol was obtained from the Local Ethics Committee. Exclusion criteria for participation involved an unstable medical condition, emergency treatment, a history of dementia, or significant visual impairment.

**Table 1:** Demographic, clinical, and disease staging factors in study group.

Parameter	All N=159	Women (n=52)	Men (n=107)	P
Age, y	66,0 (59,0 – 73,0)	70,5 (62,5 – 76,0)	64,0 (59,0 – 71,0)	0,003
No of previous vascular treatments	1,0 (0,0 – 2,0)	1,0 (0,0 – 2,0)	1,0 (0,0 – 2,0)	0,73
No of patients with amputation	13 (8%)	3 (6%)	10 (9%)	0,46
ABI of operated limb [m]	0,50 (0,225 – 0,675)	0,5 (0,2 – 0,69	0,5 (0,3 – 0,66)	0,86
ABI of non-operated limb [m]	0,69 (0,35 – 0,87)	0,78 (0,50 – 0,92)	0,6 (0,35 – 0,83)	0,14
DM, n	65 (41%)	22 (42%)	43 (40%)	0,72
HA, n	113 (71,5%)	35 (67%)	78 (73%)	0,58
CHF, n	28 (17,5%)	9 (17%)	19 (18%)	0,98
IHD, n	66 (42%)	23 (44%)	43 (40%)	0,56

Data is presented as medians and 25th and 75th quartiles or number and percentage. Significant results are marked in red.

ABI, ankle brachial-index; DM, diabetes mellitus; HA, hypertension arterialis; CHF, congestive heart failure; IHD, ischemic heart disease

The evaluation of cognitive functions was conducted using a computerized battery of cognitive tests known as the Neurotest (18). This battery comprises three main assessments:

1. Simple Reaction Test (SRT) is a psychological and neurological assessment tool used to measure an individual’s basic response time to a stimulus. This test measures the speed and accuracy of a simple reaction. Patients are required to react (press the button) as quickly as possible to the presented stimuli, in the version used these were visual stimuli. SRT is a recognized cognitive test in the population of patients with cardiovascular diseases (19)
2. Verbal Memory Test (VM): The computerized version of the test involves five consecutive administrations of a word list. In each administration, the researcher reads the same list of ten words to the participant. After each reading, the participant is asked to recall and recite as many words as possible in any order. The list remains constant across all five repetitions. During each recall attempt, three metrics are recorded: the number of correctly recalled words, the number of intrusions (incorrect words not on the list), and the number of perseverations (repeated words). To assess delayed memory, the participant is asked to recall the original list of ten words again after a 20-minute interval, this time without hearing the words repeated. The test evaluates three cognitive functions: immediate auditory memory (the number of words remembered after each reading), learning ability (the improvement in recall across the five repetitions), and delayed memory (the ability to remember the words after a delay). The test has demonstrated its effectiveness in individuals with cardiovascular diseases (20)
3. The Go/NoGo test, a classic neuropsychological assessment, evaluates functions associated with the prefrontal cortex, such as cognitive flexibility, response inhibition, and attention control. In the Neurotest cognitive battery, the Go/NoGo test involves two distinct responses: “Go” and “NoGo.” For the “Go” response, participants are instructed to press a button when a green square appears on the computer screen. Conversely, for the “NoGo” response, participants must refrain from pressing the button when a blue square appears. The stimuli, comprising 75 green squares and 25 blue squares, are presented in a random sequence. This test measures reaction time under conditions requiring cognitive control and inhibition. It evaluates the number and percentage of correct and incorrect “Go” and “NoGo” responses, as well as the reaction time (in milliseconds) for correct “Go” responses.

The Ankle-Brachial Index (ABI), a non-invasive test used to evaluate leg circulation, was conducted using a Hadeco Minidop ES100VX Doppler ultrasound device. Systolic blood pressure readings were taken from both the arms and ankles to calculate the ABI for each leg. This assessment aids in diagnosing peripheral artery disease and assessing the risk for associated complications.

Genomic DNA was extracted using the GeneMatrix Bio-Trace DNA Purification Kit from Eurx (Gdańsk, Poland) from peripheral whole blood collected with EDTA coagulant. The quantity of DNA was determined using spectrophotometry from DeNovix Inc. (Wilmington, USA). Genotyping of the BDNF-AS SNP rs6265 was carried out utilizing the TaqMan SNP Genotyping Assay with the Assay ID: C_11592758_10, in conjunction with the ViiA™ 7 Real-Time PCR System from Applied Biosystems (Carlsbad, California).

The quantification of BDNF levels was conducted using serum obtained from peripheral blood samples. The assessment followed the manufacturer’s guidelines and was carried out in duplicate with appropriately diluted serum samples. The Enzyme-Linked Immunosorbent Assay (ELISA) method was employed, beginning with the coating of a plate with a monoclonal antibody specific to BDNF (antigen). Standard solutions were used to establish a calibration curve and confirm result reliability. Subsequently, plasma samples were added to the plate and incubated for 60 minutes, allowing BDNF to bind with a specific antibody. Following the incubation period, unbound antigens were removed by washing the plate, after which an enzyme-linked antibody specific to BDNF was introduced. Another 30-minute incubation ensued, followed by plate washing to eliminate unbound antibodies. A substrate solution was then added to induce a color reaction, and the intensity of the color was measured at 450 nm, corresponding to the concentration levels of BDNF in the samples.

Statistical analyses were performed using Statistica 13.0 software. Upon assessing the data distribution via the Shapiro-Wilk test, it was determined that the data did not conform to a normal distribution pattern. Consequently, non-parametric tests were employed, and the data were primarily represented using medians, incorporating interquartile ranges (Q1-Q4). The Spearman’s rank correlation test was utilized to assess associations between variables, while differences between groups were analyzed using the Mann-Whitney U test. A significance level of p < 0.05 was chosen to establish statistical significance.

## Results

Significant differences in Neurotest results were evident between women and men (**Table 2**). Women displayed better performance on verbal memory tasks (VM2 to VM5), while men exhibited superior results in the Simple Reaction Test (SRT) and GoNoGo Test (correct Go answers). Women under 65 years old achieved better cognitive test results than women over 65 years old. Younger women acted faster in SRT and dealt with memory tasks more effective (VM3-VM5, VWMT- no answers, incorrect time, numbers of correct answers). They also had more correct Go reactions and less incorrect NoGo answers in GnG test (**Table 3**). The overall genotype frequency of BDNF Val66Met comprised 106 Met/Met or Met/Val (AS-1) and 53 Val/Val (AS-2) individuals. While no statistically significant differences were observed between AS-1 and AS-2 subgroups collectively, when these subgroups were stratified by gender, notable associations emerged (**Tables 4**, **5**). Female Met carriers demonstrated notably improved results in the VMT VM2-VM5 trials but exhibited fewer correct Go reactions in the GoNoGo test (**Table 4**). In contrast, among Val homozygous individuals, significant differences were primarily identified in the frequency of incorrect NoGo reactions, where female Val homozygotes showed a higher incidence compared to male Val homozygotes (**Table 5**). Furthermore, a positive correlation between BDNF concentration and better GoNoGo test outcomes was observed in men (0.248; p=0.01) (**Table 2**). This correlation was notably influenced by the presence of the Met allele (0.235; p=0.04). Conversely, among female Val homozygotes, a higher BDNF concentration was associated with poorer scores in VM2 and VM3 (**Table 6**).

**Table 2:** “Neurotest” cognitive battery tests results in study group.

	Female (n = 52)	Male (n = 107)	p	BDNF Val66Met	
				AS-1 n=106	AS-2 n=53
**SRT correct answers, n**	25,0 (25,0 – 25,0)	25,0 (25,0 – 25,0)	0,75	25,0 (25,0 – 25,0)	25,0 (25,0 – 25,0)
**SRT reaction time, ms**	381,4 (283,1 – 494,4)	318,5 (271,9 – 393,5)	0,02	327,9 (275,6 – 432,7)	318,5 (275,6 – 392,8)
**GnG incorrectNoGo, n**	7,0 (3,0 – 11,0)	6,0 (4,0 – 10,0)	0,79	6,0 (3,0 – 11,0)	7,0 (5,0 – 10,0)
**GnG correct Go, n**	71,0 (66,0 – 74,0)	74,0 (69,0 – 75,0)	0,01	73,0 (68,0 – 75,0)	73,0 (71,0 – 75,0)
**GnG reaction time, ms**	384,8 (325,2 – 448,4)	373,8 (338,0 – 413,8)	0,39	380,7 (341,8 – 419,2)	362,9 (333,1 – 427,6)
**VM1**	4,0 (3,0 – 5,0)	4,0 (3,0 – 5,0)	0,15	4,0 (3,0 – 5,0)	4,0 (3,0 – 5,0)
**VM2**	6,0 (5,0 – 7,0)	5,0 (5,0 – 6,0)	0,007	6,0 (5,0 – 7,0)	6,0 (5,0 – 6,0)
**VM3**	7,0 (6,0 – 8,0)	6,0 (5,0 – 7,0)	0,022	6,0 (5,0 – 7,0)	7,0 (6,0 – 7,5)
**VM4**	8,0 (6,0 – 9,0)	7,0 (6,0 – 8,0)	0,012	7,0 (6,0 – 8,0)	7,0 (6,0 – 8,0)
**VM5**	8,0 (6,0 – 9,0)	7,0 (6,0 – 8,0)	0,01	7,0 (6,0 – 8,0)	7,0 (6,0 – 8,0)
**VMDT, n**	6,0 (4,0 – 7,0)	5,0 (4,0 – 7,0)	0,091	6,0 (4,0 – 7,0)	6,0 (5,0 – 6,5)
**VWMT, no answers**	0,0 (0,0 – 1,0)	0,0 (0,0 – 1,0)	0,94	0,0 (0,0 – 1,0)	0,0 (0,0 – 0,0)
**VWMT, incorrect time**	4672 (2750 – 6515)	4187 (2454 – 5752)	0,40	4359 (2640 – 5836)	3934 (0,0 – 5752)
**VWMT, correct time**	3329 (2546 – 4282)	3429 (2625 – 4695)	0,22	3429 (2625 – 4742)	3153 (2586 – 4187)
**VWMT, n**	4,0 (2,0 – 6,0)	4,0 (2,5 – 6,0)	0,88	4,0 (2,0 – 6,0)	5,0 (3,0 – 6,5)

Data is presented as medians and 25th and 75th quartiles. Significant results are marked in red.

SRT, simple reaction test; GnG, GoNoGo test – incorrect Go answers, incorrect NoGo answers, correct Go answers, reaction VM 1-5, verbal memory trials 1,2,3,4,5; VMDT, verbal memory delayed test; VWMT, visual working memory test – number of no answers, incorrect answers time, correct answers time, number of correct answers

**Table 3:** “Neurotest” cognitive battery tests results in women broken down by gender.

	Female < 65y (n = 18)	Female >=65 (n = 34)	p
**SRT correct answers, n**	25,0 (25,0 – 25,0)	25,0 (25,0 – 25,0)	0,99
**SRT reaction time, ms**	326,1 (260,7 – 429,4)	432,2 (336,0 – 511,2)	0,043
**GnG incorrectNoGo, n**	6,0 (2,0 – 8,0)	8,0 (5,0 – 11,0)	0,09
**GnG correct Go, n**	74,0 (71,0 – 75,0)	70,0 (66,0 – 72,0)	0,01
**GnG reaction time, ms**	380,7 (341,9 – 452,3)	389,9 (325,3 – 431,0)	0,55
**VM1**	5,0 (4,0 – 5,0)	4,0 (3,0 – 5,0)	0,06
**VM2**	6,0 (6,0 – 7,0)	6,0 (5,0 – 6,0)	0,08
**VM3**	7,5 (7,0 – 8,0)	7,0 (5,0 – 8,0)	0,023
**VM4**	8,5 (8,0 – 10,0)	7,0 (6,0 – 8,0)	0,02
**VM5**	8,5 (8,0 – 10,0)	7,0 (6,0 – 8,0)	0,02
**VMDT, n**	6,5 (5,0 – 9,0)	6,0 (4,0 – 7,0)	0,14
**VWMT, no answers**	0,0 (0,0 – 0,0)	0,0 (0,0 – 1,0)	0,022
**VWMT, incorrect time**	1375 (0,0 – 4672)	5109 (3744 – 6789)	0,003
**VWMT, correct time**	3128 (2453 – 3882)	3351 (2546 – 4282)	0,75
**VWMT, n**	6,5 (5,0 – 7,0)	3,0 (1,0 – 6,0)	0,001

Data is presented as medians and 25th and 75th quartiles. Significant results are marked in red.

SRT, simple reaction test; GnG, GoNoGo test; VM 1-5, verbal memory trials 1,2,3,4,5; VMDT, verbal memory delayed test; VWMT, visual working memory test – number of no answers, incorrect answers time, correct answers time, number of correct answers

**Table 4:** Neurotest results in BDNF AS-1 group broken down by gender.

	Female (n = 33)	Male (n = 73)	p
**SRT correct answers, n**	25,0 (25,0 – 25,0)	25,0 (25,0 – 25,0)	0,23
**SRT reaction time, ms**	381,4 (291,1 – 494,4)	319,1 (269,1 – 407,4)	0,076
**GnG incorrectNoGo, n**	9,0 (5,0 – 12,0)	6,0 (3,0 – 10,0)	0,19
**GnG correct Go, n**	70,0 (66,0 – 73,0)	74,0 (69,0 – 75,0)	0,0055
**GnG reaction time, ms**	384,8 (321,4 – 430,0)	378,4 (346,1 – 406,9)	0,73
**VM1**	4,0 (4,0 – 5,0)	4,0 (3,0 – 5,0)	0,052
**VM2**	6,0 (5,0 – 7,0)	5,0 (4,0 – 6,0)	0,043
**VM3**	7,0 (6,0 – 8,0)	6,0 (5,0 – 7,0)	0,017
**VM4**	8,0 (6,0 – 9,0)	7,0 (6,0 – 8,0)	0,011
**VM5**	8,0 (6,0 – 9,0)	7,0 (5,0 – 8,0)	0,010
**VMDT, n**	6,0 (4,0 – 8,0)	5,0 (3,0 – 7,0)	0,09
**VWMT, no answers**	0,0 (0,0 – 1,0)	0,0 (0,0 – 1,0)	0,85
**VWMT, incorrect time**	4906 (2953 – 6789)	4227 (2475 – 5500)	0,35
**VWMT, correct time**	3320 (2215 – 4313)	3507 (2688 – 4835)	0,13
**VWMT, n**	4,0 (2,0 – 6,0)	4,0 (2,0 – 6,0)	0,94

Data is presented as medians and 25th and 75th quartiles. Significant results are marked in red.

SRT, simple reaction test; GnG, GoNoGo test – incorrect Go answers, incorrect NoGo answers, correct Go answers, reaction VM 1-5, verbal memory trials 1,2,3,4,5; VMDT, verbal memory delayed test; VWMT, visual working memory test – number of no answers, incorrect answers time, correct answers time, number of correct answers

**Table 5:** Neurotest results in BDNF AS-2 group broken down by gender.

	Female (n = 19)	Male (n = 34)	p
**SRT correct answers, n**	25,0 (25,0 – 25,0)	25,0 (25,0 – 25,0)	0,85
**SRT reaction time, ms**	379,2 (277,9 – 568,2)	304,8 (276,2 – 384,3)	0,29
**GnG incorrectNoGo, n**	20,0 (12,0 – 34,0)	8,5 (5,0 – 11,0)	0,043
**GnG correct Go, n**	73,0 (70,0 – 75,0)	74,0 (71,0 – 75,0)	0,51
**GnG reaction time, ms**	388,2 (337,4 – 484,6)	361,8 (333,1 – 419,8)	0,37
**VM1**	4,0 (3,0 – 5,0)	4,0 (3,0 – 5,0)	0,91
**VM2**	6,0 (6,0 – 6,0)	5,0 (5,0 – 6,0)	0,07
**VM3**	7,0 (6,0 – 7,0)	6,5 (6,0 – 8,0)	0,66
**VM4**	7,0 (6,0 – 8,0)	7,0 (6,0 – 8,0)	0,36
**VM5**	7,0 (6,0 – 8,0)	7,0 (6,0 – 8,0)	0,36
**VMDT, n**	6,0 (4,0 – 7,0)	5,0 (5,0 – 6,0)	0,28
**VWMT, no answers**	0,0 (0,0 – 1,0)	0,0 (0,0 – 0,0)	0,95
**VWMT, incorrect time**	4516 (0,0 – 6515)	3794 (0,0 – 5756)	0,82
**VWMT, correct time**	3448 (2625 – 3828)	3130 (2532 – 4187)	0,98
**VWMT, n**	4,0 (3,0 – 7,0)	5,0 (4,0 – 6,0)	0,93

Data is presented as medians and 25th and 75th quartiles. Significant results are marked in red.

SRT, simple reaction test; GnG, GoNoGo test – incorrect Go answers, incorrect NoGo answers, correct Go answers, reaction VM 1-5, verbal memory trials 1,2,3,4,5; VMDT, verbal memory delayed test; VWMT, visual working memory test – number of no answers, incorrect answers time, correct answers time, number of correct answers

**Table 6:** Correlations of BDNF’s serum level with Neurotest cognitive results in study group.

	ALL group (n=159)		BDNF AS-1 (n=106)		BDNF AS-2 (n=53)	
	Female (n = 52)	Male (n = 107)	Female (n = 33)	Male (n = 73)	Female (n = 19)	Male (n = 34)
**SRT correct answers, n**	0,125; p=0,37	-0,024; p=0,80	-0,108; p=0,54	-0,021; p=0,86	-0,056; p=0,81	0,057; p=0,74
**SRT reaction time, ms**	0,143; p=0,31	-0,144; p=0,13	0,066; p=0,71	-0,118; p=0,32	0,241; p=0,32	-0,273; p=0,11
**GnG incorrectNoGo, n**	0,139; p=0,32	-0,134; p=0,16	0,034; p=0,85	-0,076; p=0,52	0,271; p=0,26	-0,271; p=0,12
**GnG correct Go, n**	0,086; p=0,54	0,248; p=0,01	0,179; p=0,31	0,235; p=0,04	-0,104; p=0,67	0,341; p=0,04
**GnG reaction time, ms**	0,082; p=0,56	-0,056; p=0,56	-0,142; p=0,43	-0,127; p=0,28	0,399 p=0,09	0,001; p=0,99
**VM1**	-0,198; p=0,15	-0,013; p=0,89	-0,115; p=0,52	-0,084; p=0,47	-0,300; p=0,21	0,164; p=0,35
**VM2**	-0,097; p=0,49	0,047; p=0,63	0,073; p=0,68	0,158; p=0,18	-0,456; p=0,04	-0,192; p=0,27
**VM3**	-0,027; p=0,84	0,021; p=0,83	0,219; p=0,22	-0,027; p=0,38	-0,483; p=0,03	0,153; p=0,38
**VM4**	-0,171; p=0,22	0,173; p=0,07	-0,052; p=0,77	0,077; p=0,51	-0,367; p=0,12	0,475; p=0,004
**VM5**	-0,172; p=0,22	0,180; p=0,06	-0,053; p=0,76	0,088; p=0,45	-0,377; p=0,11	0,480; p=0,004
**VMDT, n**	-0,185; p=0,18	0,099; p=0,31	-0,301; p=0,08	0,099; p=0,40	0,089; p=0,71	0,171; p=0,33
**VWMT, no answers**	0,089; p=0,53	-0,123; p=0,20	0,051; p=0,77	-0,101; p=0,39	0,132; p=0,59	-0,216; p=0,21
**VWMT, incorrect time**	0,099; p=0,48	-0,110; p=0,25	-0,092; p=0,61	-0,046; p=0,69	0,416; p=0,07	-0,371; p=0,03
**VWMT, correct time**	-0,046; p=0,74	0,039; p=0,69	0,128; p=0,47	-0,084; p=0,47	-0,466; p=0,04	0,306; p=0,07
**VWMT, n**	0,101; p=0,47	0,124; p=0,12	0,234; p=0,49	0,038; p=0,74	-0,086; p=0,72	0,365; p=0,03

Data is presented as Spearmen r correlation factor as p – significance coefficient. Significant results are marked in red.

SRT, simple reaction test; GnG, GoNoGo test – incorrect Go answers, incorrect NoGo answers, correct Go answers, reaction VM 1-5, verbal memory trials 1,2,3,4,5; VMDT, verbal memory delayed test; VWMT, visual working memory test – number of no answers, incorrect answers time, correct answers time, number of correct answer

## Discussion

Numerous studies have already shown relationships between cardiovascular diseases and cognitive dysfunctions (21). Such correlations were notice also in CLI. Patients with symptomatic CLI occurred to have cognitive decline (22–24).

This study also focuses on patients diagnosed with CLI and examines their neurocognitive functioning. The investigation delves into the interplay between collected neurocognitive data, serum levels of BDNF, and the Val66Met BDNF polymorphism. Notably, our study unveils gender-specific correlations of the BDNF Val66Met polymorphism with various neurocognitive features, marking a novel contribution within the CLI patient population.

Initially, our findings revealed a notable superiority in short-term verbal memory among women, demonstrated by a higher recall of words in attempts 2-5 of the VMT. This aligns with existing literature indicating women’s adeptness in handling verbal memory tasks compared to men (25). In addition, in the study group women were older than men (p=0,003). The median age of patients was 70,5 years in females and 64 years in males (**Table 1**). Age is the most obvious factor associated with cognitive deterioration (26). In that context, the fact that examined women, despite their older average age, still performed better on memory tests than men, makes our results more significant. Also it is not surprising that women over 65 years old accomplished worse scores in various cognitive tasks such as SRT, VMT, VWMT and GnG test than women under 65 years old.

Conversely, our study revealed that men exhibited superior executive control, as evidenced by their performance in the SRT and GoNoGo tasks. It’s widely acknowledged that men tend to have faster reaction times (27). However, while executive control tests like GoNoGo often display male predominance, previous research lacks a clear consensus on this aspect. For instance, Sjoberg and Cole observed that women outperformed men in No-Go trials, with no discernible difference in reaction times during Go trials (25). Moreover, some studies indicated slower and more inconsistent reactions for both genders during the TAP Go/NoGo paradigm (28). Conversely, other researchers did not identify any sex-based differences in this test (29). Given the intricate neural networks involved in cognitive control, interpreting sex differences solely based on a simplistic tool such as the GoNoGo test remains challenging.

Numerous studies have established a connection between the Val66Met polymorphism and cognitive performance. In the general population, the presence of the Met allele has been associated with lower scores in cognitive tests, particularly in declarative memory tasks (30) and executive functions (31). Moreover, the Met allele has been linked to poorer outcomes in structural and functional MRI analyses of brain regions, notably the prefrontal cortex and hippocampus (32, 33), and has even been implicated in neurodegenerative diseases characterized by cognitive decline, such as Alzheimer’s disease (34). According to the metanalysis of Yi Long Toh et al., including patients with cardiovascular diseases, other disease states and healthy people, Val/Val homozygous did better in tasks concerned the memory, while Met carriers performed better in the field of executive function, but the influence of Met allele has not been clearly determined (35). On the other hand, in the population of patients with cardiovascular diseases, Szabo et al. found out that subjects with one or more Met alleles had better language and visuospatial abilities, memory, attention and executive functions than Val/Val carriers (36). In our study Met allele also seemed to improve cognitive functioning, an interesting distinction emerged between subgroups AS-1 and AS-2. Women carrying the Met allele exhibited superior memory test performance compared to men, a correlation not observed in Val homozygotes. This observation suggests that despite the well-established negative influence of the Met allele on cognitive function, its impact might be less pronounced among women or potentially display a protective effect. However, the influence of the BDNF Val66Met polymorphism on cognition may exhibit sex-specific patterns. For instance, Laing et al. described the association between Val66Met polymorphism and perceptual speed in females (37). Our analyses did not show significant differences between abilities in the field of perceptual speed. In turn, Foltynie et al. observed that female Parkinson’s disease patients, possessing Met alleles and displaying lower BDNF secretion rates, demonstrated superior executive functioning (38). In our study, a similar trend was noticed, indicating a potentially protective role of the Met allele in memory preservation among women (manifested in VM2-VM5 attempts), while this correlation was not evident in men. These findings suggest a potential sex-dependent impact of the Val66Met polymorphism on brain regions like the prefrontal cortex or hippocampus. Nonetheless, our study lacked neuroimaging protocols, which limited our ability to identify structural pathologies that might contribute to deficits in executive functioning and memory.

Some other researches reported an important reduction in plasma BDNF levels in woman (39). On the other hand, animal studies have indicated that estrogen can elevate BDNF levels (40). Therefore, in our investigation, female patients with CLI might exhibit higher BDNF levels than males, particularly noticeable in the Met carriers group where BDNF levels are typically lower, but our statistical analysis did not confirm the importance of results in BDNF serum levels depending on gender.

Furthermore, current findings suggest the involvement of BDNF in dopaminergic pathways through dopamine D3 receptors (41). In animal models, activation of these receptors has been linked to inhibited working memory ability. Similarly, human studies have shown that Met carriers with Parkinson’s disease performed better on working memory tasks (41). This association might explain the negative correlation observed between BDNF levels and memory test scores presented in **Table 6**. However, elucidating these mechanisms requires further investigation.

Beyond the influence of BDNF gene variants, our study revealed that men outperformed women in the GoNoGo test. Moreover, the higher the serum level of BDNF was the more correct GnG Go answers were. The male advantage in this test consisted of an increased number of correct Go responses in the group of male Met carriers and an increased number of incorrect NoGo responses in the group of female Val homozygotes. Also literature pointed out go/no-go scores as significantly lower among the subjects with the Val/Val genotype than among the Met carriers for example in Alzheimer disease (42).

In summary, our study underscores that BDNF serum concentration and its polymorphism exert an influence on cognitive processes in a sex-dependent manner.

## Data Availability

The original contributions presented in the study are included in the article/supplementary material. Further inquiries can be directed to the corresponding author.
